# GLP-1 receptor agonists for Alzheimer’s disease: Lessons from trials and translational challenges

**DOI:** 10.1016/j.xcrm.2026.102918

**Published:** 2026-08-18

**Authors:** Ramona Stringhi, Alessandro Padovani, Elena Marcello

**Affiliations:** 1 Department of Pharmacological and Biomolecular Sciences “Rodolfo Paoletti”, University of Milan, 20133 Milan, Italy; 2 Neurology Unit, Department of Clinical and Experimental Sciences, University of Brescia, 25123 Brescia, Italy; 3 Department of Continuity of Care and Frailty, Neurology Unit, ASST Spedali Civili Hospital, 25123 Brescia, Italy; 4 Neurobiorepository and Laboratory of Advanced Biological Markers, University of Brescia and ASST Spedali Civili Hospital, 25123 Brescia, Italy; 5 Brain Health Center, University of Brescia, 25123 Brescia, Italy

## Abstract

Alzheimer’s disease (AD) is a multifactorial disorder that requires combined therapeutic strategies beyond amyloid clearance. Although GLP-1 receptor agonists show strong mechanistic rationale and supportive epidemiological signals for neuroprotection, large randomized trials in symptomatic AD have yielded negative clinical outcomes and do not support the therapeutic use in AD.

## Main text

Alzheimer’s disease (AD) is the leading cause of dementia, responsible for 60–80% of cases and is marked by progressive cognitive decline leading to severe dementia and premature death. Its neuropathological hallmarks include amyloid β (Aβ) plaques and tau-containing neurofibrillary tangles associated with neuron loss.^1^ AD prevalence rises with age, and global incidence is increasing due to population aging and longer life expectancy, making it a growing global challenge for patients, families, and healthcare systems.

For decades, the pharmacological management of AD has relied on acetylcholinesterase inhibitors and memantine, which provide modest symptomatic benefit without meaningfully altering disease progression. Advances in the biological understanding of AD pathogenesis have enabled a paradigm shift toward disease-modifying therapies and biomarker-driven definitions of the AD continuum. The staging criteria classify individuals based on the presence of Aβ, tau, and neurodegeneration, as quantified using positron emission tomography (PET) and cerebrospinal fluid (CSF) measures.^1^ More recently, phosphorylated tau at amino acid 217 (p-tau217) has emerged as a highly sensitive and specific AD blood biomarker.^1^ In parallel, the clinical introduction of amyloid-lowering monoclonal antibodies (mAbs) for early symptomatic AD represents a conceptual shift toward therapies that directly target core pathogenic mechanisms rather than solely addressing symptoms.^1^ Even though mAbs designate a major advance in targeting AD, their practical use is limited by heterogeneous responses and the risk of amyloid-related imaging abnormalities (ARIAs), which necessitate routine MRI monitoring and careful risk stratification, particularly according to APOE ε4 status.^2^

Therefore, substantial efforts are still required to identify novel pharmacological targets and complementary therapeutic strategies able to offer greater efficacy, safety, convenience and accessibility for AD patients.

Therapeutic advances beyond amyloid will require reframing AD as a multifactorial disorder shaped by the convergence of diverse, interconnected biological processes.^1^ While Aβ and tau pathology remain central to disease pathogenesis, a broad spectrum of additional mechanisms contributes to disease onset and progression, including synaptic failure, chronic neuroinflammation, impaired cerebral energy metabolism, insulin resistance, mitochondrial dysfunction, defective autophagy, and dysregulated oxidative stress responses.^1^ Hence, effective therapeutic intervention will likely require integrated, multimodal strategies capable of concurrently targeting multiple pathological pathways.

In this framework, the glucagon-like peptide-1 receptor agonists (GLP-1RAs) emerge as particularly attractive candidates, given their pleiotropic effects across multiple biological pathways implicated in AD.^3^

GLP-1RAs are a class of pharmacological agents developed to improve glycemic control and reduce energy intake by mimicking the actions of the endogenous incretin hormone glucagon-like peptide-1 (GLP-1), which is secreted by enteroendocrine L-cells of the distal small intestine and colon in response to nutrient intake.^3^ Peripherally, GLP-1 enhances glucose-dependent insulin secretion, suppresses glucagon, delays gastric emptying, and lowers fasting and postprandial glycemia, thereby regulating glucose homeostasis and food intake.^3^

The physiologically synthesized GLP-1 has a very short plasma half-life due to its rapid degradation by dipeptidyl peptidase-4 (DPP-IV) and its consequent clearance. To overcome this point, long-acting peptide analogs with enhanced stability and prolonged biological activity have been developed.^3^

GLP-1RAs are clinically approved for the treatment of type 2 diabetes mellitus (T2DM) and obesity.^3^^,^^4^ Epidemiological studies show that T2DM increases the risk of AD across diverse populations, suggesting that impaired insulin signaling contributes to AD pathogenesis by enhancing oxidative stress, mitochondrial dysfunction, neuroinflammation, and the accumulation of tau and Aβ.^3^

This evidence supported the potential repurposing of GLP-1RAs for AD therapy. In addition, beyond their role in glycemic control, GLP-1RAs exert pleiotropic effects in multiple organs, including the brain.^3^ Notably, GLP-1 is synthesized in the central nervous system by preproglucagon-expressing neurons in the brainstem, and GLP-1 receptors (GLP-1Rs) are widely expressed on neurons and glial cells in AD-relevant regions such as the hippocampus, cortex, hypothalamus, and basal ganglia.^4^ Therefore, this broad receptor distribution highlights the presence of an intrinsic GLP-1 signaling network operating directly in the central nervous system (CNS).

Several GLP-1RAs have been shown to cross the blood-brain barrier (BBB) to varying degrees^5^ and have been reported, primarily in preclinical models and early clinical studies, to suppress pathological protein aggregation, attenuate neuroinflammatory responses, reduce oxidative stress, preserve mitochondrial function, upregulate neurotrophic factors, limit neurodegeneration, and promote neuronal survival and regeneration.^3^ Notably, many of these effects intersect with key pathological mechanisms in AD and arise from GLP-1R signaling.^3^^,^^4^ The downstream signaling cascades engaged by GLP-1R activation and their relevance to neurodegeneration have been comprehensively reviewed^3^ and are therefore only briefly summarized here. Activation of the Gα-coupled GLP-1R stimulates adenylate cyclase, leading to an increase in intracellular cyclic adenosine monophosphate (cAMP). This rise in cAMP is essential for activating key cellular effectors, including protein kinase A (PKA) and exchange proteins directly activated by cAMP (Epac). These effectors, in turn, initiate downstream signaling pathways that extend beyond metabolic regulation and are directly implicated in neurodegenerative processes. A key consequence of GLP-1R activation is the recruitment of the PI3K-Akt signaling axis, which promotes neuronal survival and limits apoptotic signaling in preclinical models. In parallel, GLP-1R stimulation has been shown, in specific neural contexts, to activate MAPK/ERK pathways, contributing to neurotrophic responses and synaptic maintenance. Moreover, GLP-1R-mediated signaling converges on the activation of the cAMP response element-binding protein (CREB), a key transcriptional regulator of synaptic plasticity and memory. CREB activation downstream of GLP-1R signaling induces the expression of brain-derived neurotrophic factor (BDNF) thereby fostering neurogenesis, synaptic plasticity, and cognitive function in experimental models. Additional downstream effects of GLP-1R activation include Akt-dependent inhibition of glycogen synthase kinase-3β (GSK-3β), which has been associated with β-catenin stabilization and transcriptional programs implicated in neuronal resilience and energy homeostasis. Notably, GSK-3β also plays a central role in tau phosphorylation and the formation of neurofibrillary tangles. Furthermore, GLP-1R signaling has been reported to intersect with cellular energy-sensing pathways, such as AMP-activated protein kinase (AMPK) and mechanistic target of rapamycin (mTOR), thereby influencing mitochondrial function, redox balance, and cellular stress responses in the brain. Collectively, these central signaling mechanisms converge on key pathological processes underlying AD, including synaptic dysfunction, impaired neurotrophic support, metabolic stress, and neuronal loss, providing a strong mechanistic rationale for the repurposing of GLP-1RAs as potential therapies in AD.

In addition, clinical and epidemiological evidence suggested that treatment with GLP-1RAs might reduce the risk of dementia in patients with T2DM.^3^ Based on the protective effects, numerous clinical trials have been conducted or are currently ongoing in AD. In Table 1, we summarize the available clinical evidence, organized by individual GLP-1RAs. Of the GLP-1RAs approved for T2DM, only exenatide, liraglutide, and semaglutide have been investigated in clinical trials in patients with AD.

**Table 1:** Design and outcomes of clinical trials evaluating GLP-1RAs in Alzheimer’s disease

Clinical Trial	Drug	Inclusion criteria	Dose and Duration	Primary endpoint	Primary endpoint met	Secondary endpoints	Secondary endpoints met	Reference
**EVOKE,** **EVOKE+ (NCT04777396, NCT04777409)**	semaglutide	clinical stage: MCI or mild AD; neuropsychological assessment: yes; AD biomarker confirmation: yes; metabolic criteria: no	oral, once daily, titrated up to 14 mg; 156 weeks	change in CDR-SB from baseline to week 104	**no**	change in ADCS-ADL-MCI score from baseline and time to progression to a CDR global score of ≥1.0 among participants with a CDR global score of 0.5 at baseline, both assessed at week 104	**no** (but improvement in AD biomarkers reported)	Cummings et al.^6^
**NCT05891496**	semaglutide	clinical stage: MCI or mild AD; neuropsychological assessment: yes; AD biomarker confirmation: yes; metabolic criteria: no	SC, weekly, titrated up to 1.0 mg; 12-week DB + 52-week OLE	gene expression in CSF and blood (scRNA-seq)	**exploratory**	PK analysis; safety and tolerability	**yes**	Mystkowski et al.^7^
**NCT06072963**	semaglutide ± intranasal insulin	clinical stage: MCI; neuropsychological assessment: yes; AD biomarker confirmation: no; metabolic criteria: yes	oral, once daily, titrated up to 14 mg ± intranasal insulin; 12 months	change in composite cognitive *Z* score from baseline to month 6 and 12; change in cerebral blood flow (ASL-MRI) and glucose metabolism ([^1^^8^F]FDG-PET) at 6 months	**currently recruiting**	cognitive and functional evaluation; neuroimaging; blood-based biomarkers; insulin signaling in brain-derived EVs.	**currently recruiting**	Davidy et al.^8^
**NCT01469351**	liraglutide	clinical stage: mild to moderate AD; neuropsychological assessment: yes; AD biomarker confirmation: no; metabolic criteria: no	SC; once daily, titrated up to 1.8 mg; 26 weeks	change in cerebral amyloid ([^11^C]PiB-PET) from baseline to week 26	**no**	cerebral glucose metabolism ([^1^^8^F]FDG-PET); cognitive evaluation	**partially** (glucose metabolism stabilization only)	Gejl et al.^9^
**ELAD (NCT01843075)**	liraglutide	clinical stage: mild to moderate AD; neuropsychological assessment: yes; AD biomarker confirmation: no; metabolic criteria: no	SC; once daily 1.8 mg; 52 weeks	change in cerebral glucose metabolism ([^1^^8^F]FDG-PET) from baseline to week 52	**no**	change in ADAS-Exec, CDR-SB, ADCS-ADL score; MRI; amyloid load and tau deposition; microglial activation; safety	**partially** (slower change in ADAS-Exec score; MRI outcomes preserved; acceptable safety profile)	Edison et al.^10^
**NCT01255163**	exenatide	clinical stage: MCI or mild AD; neuropsychological assessment: yes; AD biomarker confirmation: yes; metabolic criteria: no	SC; twice daily titrated up to 10 μg; 18 months	safety and tolerability	**yes**	change in MMSE, ADAS-Cog, CDR-SB, CDR global score; CSF biomarkers; MRI	**no** (except for a reduction of Aβ_42_ in neuronal-derived EVs)	Mullins et al.^11^
**NCT02847403**	long-acting exenatide	clinical stage: MCI; neuropsychological assessment: yes; AD biomarker confirmation: no; metabolic criteria: yes	SC; once weekly 2 mg; 32 weeks	change in ADAS-Cog score from baseline to week 16 and 32	**no** (sex-specific worsening in females)	neuropsychological assessments; MRI	**no**	Dei Cas et al.^12^

Abbreviations: MCI, mild cognitive impairment; AD, Alzheimer’s disease; CDR-SB, Clinical Dementia Rating - Sum of Boxes; ADCS-ADL-MCI, Alzheimer's Disease Cooperative Study Activities of Daily Living - MCI; SC, subcutaneous; DB, double-blind; OLE, Open-Label Extension; CSF, cerebrospinal fluid; scRNA-seq, single-cell RNA sequencing; PK, pharmacokinetics; ASL-MRI, Arterial Spin Labeling - magnetic resonance imaging; [^18^F]FDG-PET, Fluorine-18 Fluorodeoxyglucose - Positron Emission Tomography; EVs, extracellular vesicles; [^11^C]PiB-PET, Carbon-11-labeled Pittsburgh compound B Positron Emission Tomography; ADAS-Exec, Alzheimer’s Disease Assessment Scale-Executive domain; MMSE, Mini-Mental State Examination; ADAS-Cog, Alzheimer’s Disease Assessment Scale-Cognitive Subscale.

Exenatide is the first GLP-1RA developed in 1995 and approved in 2005 and shares 53% sequence identity with human GLP-1. Liraglutide and semaglutide exhibit greater structural homology and enhanced stability through specific amino acid substitutions and lipid side-chain modifications that prolong activity and reduce DPP-IV degradation. Liraglutide incorporates a C16 fatty acid chain, while semaglutide introduces an α-aminoisobutyric acid substitution and a C18 fatty acid chain, along with PEG modification, conferring increased half-life and albumin binding capacity for once-weekly dosing.^3^

Preclinical evidence supported the potential utility of GLP-1RAs in AD. In AD mouse models, liraglutide prevents memory deficits, preserves hippocampal synaptic structure and plasticity, reduces Aβ aggregation, and attenuates cortical and hippocampal neuroinflammation, while also promoting neurogenesis. Consistent with these findings, exenatide demonstrates comparable neuroprotective effects in AD models, including reduced amyloid burden, preservation of synaptic integrity, mitigation of oxidative stress, and improvement of mitochondrial dysfunction, highlighting the neuroprotective potential in preclinical AD.^3^

Exenatide was the first GLP-1RA evaluated in clinical trials involving individuals with mild AD and mild cognitive impairment (MCI). The phase 2 trial demonstrated that exenatide was safe and well tolerated but showed no significant cognitive, imaging, or biomarker benefits despite a decrease in Aβ in plasma extracellular vesicles enriched for neuronal origin.^11^ The subsequent phase 3 trial in individuals with MCI and prediabetic dysglycemia similarly found no cognitive improvement with long-acting exenatide, aside from expected metabolic effects.^12^

Regarding liraglutide, the first clinical trial evaluated 38 patients with mild to moderate AD. Over 26 weeks, liraglutide treatment prevented the decline in cerebral glucose metabolism observed in the placebo group and significantly increased blood-brain glucose transfer capacity in the cerebral cortex, suggesting restoration of glucose transport across the BBB. Therefore, while liraglutide appeared to stabilize cerebral metabolic decline relative to placebo, the study was underpowered to detect differences in cognition or amyloid load.^9^ Nevertheless, this early proof-of-concept clinical investigation provided mechanistic evidence supporting a central metabolic effect of liraglutide in AD, but its limited sample size and short duration precluded definitive efficacy conclusions. However, it helped justify subsequent larger investigations such as the ELAD phase 2b trial, which aimed to assess longer-term effects on metabolism and clinical outcomes in a larger cohort. The ELAD study was a multicenter, randomized, double-blind, placebo-controlled phase 2b trial designed to assess the effects of the GLP-1RA liraglutide in individuals with mild AD.^10^ The primary endpoint was the change in cerebral glucose metabolic rate, while the key secondary outcomes comprised changes in cognitive performance**.** Results from the completed trial indicated no statistically significant difference in the primary outcome, with only a small and non-significant between-group difference in cerebral glucose metabolism at 52 weeks.^10^ Liraglutide was generally safe and well tolerated in this non-diabetic AD population. Secondary analyses suggested modest cognitive benefits, though these findings were exploratory. No significant differences were seen on measures such as the Clinical Dementia Rating-Sum of Boxes. Overall, the ELAD trial did not demonstrate a clear advantage in the prespecified primary endpoint, highlighting the complexity of translating mechanistic rationale into clinically meaningful outcomes.

Among GLP-1RAs, semaglutide has been evaluated most extensively in AD through real-world epidemiology, large phase 3 outcome trials, and mechanistic and exploratory clinical studies. An emulated trial using a nationwide electronic health record database of over 1 million individuals with T2DM and no prior AD diagnosis reported that semaglutide use was associated with a substantially lower risk (≈40–70%) of first-time AD diagnosis compared with multiple other antidiabetic therapies, with consistent effects across age, sex, and obesity subgroups.^13^ In contrast to these observational findings, the phase 3 EVOKE and EVOKE+ randomized, double-blind, multi-country, placebo-controlled trials, enrolling 3,808 participants with biomarker-positive AD in the MCI or mild dementia stage receiving oral semaglutide, in addition to standard therapy, up to three years, failed to meet their primary endpoint of slowing clinical progression.^6^ The two trials were identical in design except that EVOKE+ permitted inclusion of a small subset of participants with concomitant small-vessel pathology. Despite negative clinical outcomes, a CSF substudy of the EVOKE trials showed that semaglutide was associated with modest (approximately 10%) reductions in several AD-related biomarkers, including p-tau217 and markers of neuroinflammation, neurodegeneration, and synaptic function (YKL-40, total tau, and neurogranin). These effects are smaller than the 25% reductions observed with anti-amyloid mAbs and may reflect limited CNS bioavailability. However, in the absence of demonstrated clinical benefit, such modest biomarker shifts should be interpreted cautiously and do not, by themselves, establish meaningful disease modification, although they may suggest limited engagement of AD-related biological pathways.^6^ Beyond these important trials, smaller hypothesis-driven studies continue to explore the biological effects of semaglutide in AD. A mechanistic trial employing once-weekly subcutaneous semaglutide demonstrated the feasibility of interrogating central and peripheral immune and molecular signatures, including transcriptomic changes in CSF and blood.^7^ Furthermore, a pilot combination trial pairing oral semaglutide with intranasal insulin is assessing feasibility, safety, cognitive outcomes, and multimodal biomarkers in older adults with MCI and metabolic syndrome.^8^

**Figure 1:**
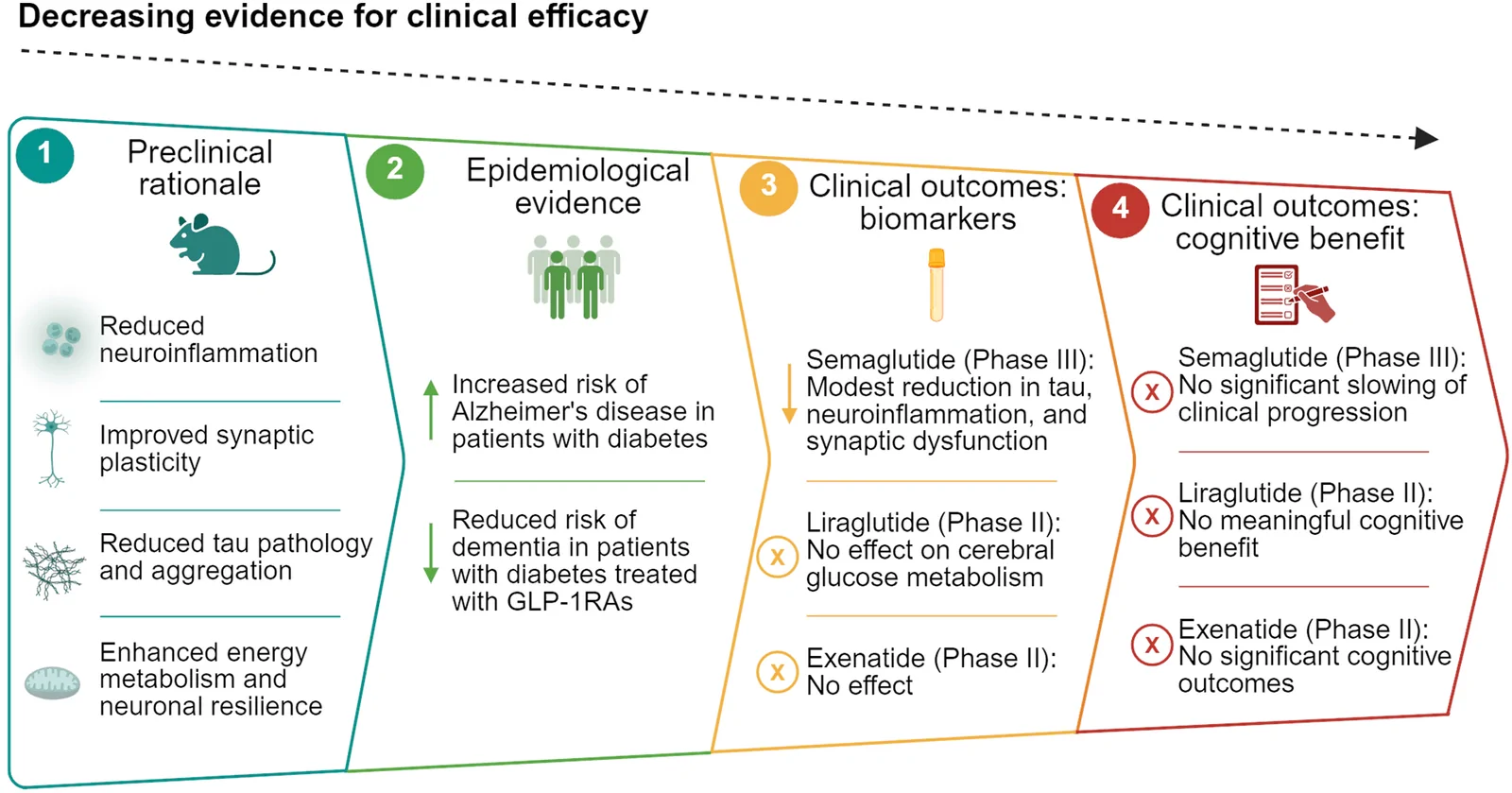
Translational landscape of GLP-1RAs in AD Preclinical evidence suggests that GLP-1RAs exert multiple neuroprotective effects, including reduced neuroinflammation and tau aggregation, improved synaptic plasticity, and enhanced neuronal survival and metabolic resilience. Epidemiological studies have consistently reported an increased risk of AD in individuals with T2DM, while observational data associate GLP-1RAs use with a lower risk of dementia. Together, these preclinical and epidemiological findings provided the rationale for the development of clinical trials investigating GLP-1RAs as potential disease-modifying therapies for AD. Human biomarker studies have subsequently shown heterogeneous and generally modest signals of biological activity, including effects on CSF biomarkers related to tau pathology, neuroinflammation, synaptic dysfunction, and cerebral glucose metabolism across different compounds. However, despite encouraging mechanistic and epidemiological signals, randomized clinical trials conducted to date have not demonstrated significant clinical efficacy or meaningful slowing of cognitive decline in AD. The narrowing funnel and descending arrow illustrate the progressive reduction in translational strength from mechanistic rationale to clinical outcomes, emphasizing the gap between preclinical promise and therapeutic benefit in AD. Green boxes indicate strong and consistent supportive evidence; yellow boxes represent moderate or mixed findings; and red boxes denote limited, negative, or inconclusive clinical evidence.

Despite the abundance of preclinical studies supporting the use of GLP-1RAs in AD, as well as observational analyses in T2DM patients suggesting a reduced risk of dementia among those treated with semaglutide, clinical trials in patients with AD have not demonstrated a significant effect on disease progression (Figure 1). These findings highlight the translational gap between preclinical models, which do not fully capture the complexity and spectrum of pathological changes observed in the human disease, and the lack of clinical efficacy in established AD. They also underscore the need to interpret observational associations cautiously, given the potential for confounding factors inherent in real-world datasets.

Furthermore, several key questions remain unresolved. One critical issue is whether GLP-1RAs efficacy relies on crossing brain barriers to engage central receptors or on peripheral metabolic optimization. While GLP-1RAs like liraglutide penetrates the BBB, their neuroprotective effects may also stem from systemic improvements, such as reduced inflammation, enhanced insulin sensitivity, and gut-brain axis activation. For instance, in the EVOKE trial, semaglutide treatment was associated with significantly reduced plasma high-sensitivity C-reactive protein concentrations, indicating a potential role in reducing peripheral inflammation.^6^ Differentiating between these direct central effects and indirect peripheral benefits is essential for refining drug design and dosing strategies.^5^ Of note, BBB integrity in AD is influenced by multiple metabolic and genetic factors. This variability may contribute to a heterogeneous effect of GLP-1RAs dependent on BBB integrity, which is potentially more pronounced in APOE ε4 carriers with insulin-resistance.^14^ It is imperative that the role of BBB integrity and its association with genetic and metabolic factors be definitively explored in forthcoming trials. This exploration will have far-reaching implications that extend beyond the APOE-related mechanisms known to be associated with different AD subtypes.^15^

In addition, the interpretation of these clinical trials is further complicated by methodological challenges, most notably the issue of “functional unblinding.” The active treatment arms in GLP-1RAs trials frequently experience distinct gastrointestinal side effects and significant weight loss, which contrasts sharply with the placebo group. This physical discrepancy allows participants and investigators to infer group assignment, potentially biasing subjective cognitive assessments and questioning whether the control arm serves as a true placebo. Furthermore, although the EVOKE trials expanded safety data for semaglutide in an older and relatively frail AD population,^6^ its safety in non-diabetic elderly patients warrants careful scrutiny. Unlike the obese, diabetic populations for which these agents were developed, patients with AD are often frail; therefore, GLP-1RAs-induced weight loss may be detrimental rather than beneficial.

Finally, the stark contrast between the robust protective effects seen in real-world diabetic registries and the negative results of clinical trials in symptomatic AD suggests a fundamental distinction between neuroprotection and therapeutic treatment. The registry data may reflect a preventative trajectory, where chronic GLP-1RAs exposure mitigates metabolic risk factors years before neurodegeneration becomes irrevocable. In contrast, initiating treatment in established AD, as in the EVOKE trials, may face the hurdle of irreversible synaptic and neuronal loss. Consequently, GLP-1RAs may be better positioned as neuroprotective or risk-modifying agents rather than as therapeutic interventions for established dementia. Their potential utility may lie in early intervention strategies targeting high-risk individuals with metabolic dysregulation, bridging the gap between metabolic health and cognitive resilience. Within this framework, APOE ε4 carriers may represent a biologically relevant subgroup for future studies. APOE ε4 confers vulnerability not only through Aβ-related mechanisms, but also through metabolic and neurovascular pathways. APOE ε4 genotype is associated with impaired insulin signaling, altered cerebral glucose utilization, endothelial dysfunction, BBB disruption, and increased susceptibility to vascular brain injury.^14^^,^^15^ These alterations could hypothetically amplify the detrimental impact of T2DM, insulin resistance, obesity, and small-vessel disease on AD progression. GLP-1RAs, by improving systemic insulin sensitivity, reducing metabolic inflammation, promoting weight loss, enhancing vascular function, and potentially modulating brain energy metabolism and neuroinflammation, could theoretically buffer the metabolic-vascular phenotype associated with APOE ε4. Accordingly, APOE ε4 status should be considered not only as a marker of risk and safety, but also as a potential predictor of differential response to metabolic–neurovascular interventions, although this hypothesis remains to be tested in future studies. In this perspective, GLP-1RAs could therefore be explored as preventive or risk-modifying interventions targeting metabolic vulnerability, rather than as disease-modifying treatments for symptomatic AD.

## Acknowledgments

E.M. received funding from Italian Ministry of Research and University (MUR) (PRIN2022 PNRR P2022R2E8N) and from the Giovanni Armenise Harvard Foundation and AIRALZH ONLUS (2023 Armenise Harvard-AIRALZH Mid-Career Award in Neurodegenerative Diseases - AHA MCA).

A.P. has been supported by grants of the Italian Ministry of University and Research PRIN COCOON (2017MYJ5TH) and PRIN 2021 RePlast (20202THZAW), Prin 2022 EGADi (P2022TKN8C), the H2020 IMI IDEA-FAST (ID853981), #NEXTGENERATIONEU (NGEU) funded by the Ministry of University and Research, National Recovery and Resilience Plan (NRRP), project MNESYS (PE0000006) – a multiscale integrated approach to the study of the nervous system in health and disease (DN. 1553 11.10.2022) – subproject DIGI-BRAIN Grant/Award Numbers RF-2018-12366209 and RF-2019-12369272, and PNRR-Health PNRR-MAD-2022-12376110, the Next Generation EU - NRRP M6C2 - Investment 2.1 Enhancement and strengthening of biomedical research in the NHS- PNRR – PNRR PNRR-Health PNRR-MAD-2022-12376110.

Figure 1 was created with BioRender.com.

## Declaration of interests

E.M. received speaker fees from Eli Lilly and GE Healthcare, advisory board fees from Roche, and teaching fees from Eisai. A.P. received personal compensation as a consultant/scientific advisory board member for Biogen, Eisai Eli Lilly, General Healthcare (GE), Lundbeck, Nestlé, and Roche.

## Declaration of generative AI and AI-assisted technologies in the writing process

During the preparation of this work, the authors used ChatGPT to improve language and readability. After using this tool, they reviewed and edited the content as needed and take full responsibility for the publication’s content.
